# Transcriptomic Analysis of (Group I) *Clostridium botulinum* ATCC 3502 Cold Shock Response

**DOI:** 10.1371/journal.pone.0089958

**Published:** 2014-02-24

**Authors:** Elias Dahlsten, Marita Isokallio, Panu Somervuo, Miia Lindström, Hannu Korkeala

**Affiliations:** Department of Food Hygiene and Environmental Health, Faculty of Veterinary Medicine, University of Helsinki, Helsinki, Finland; Institute Pasteur, France

## Abstract

Profound understanding of the mechanisms foodborne pathogenic bacteria utilize in adaptation to the environmental stress they encounter during food processing and storage is of paramount importance in design of control measures. Chill temperature is a central control measure applied in minimally processed foods; however, data on the mechanisms the foodborne pathogen *Clostridium botulinum* activates upon cold stress are scarce. Transcriptomic analysis on the *C. botulinum* ATCC 3502 strain upon temperature downshift from 37°C to 15°C was performed to identify the cold-responsive gene set of this organism. Significant up- or down-regulation of 16 and 11 genes, respectively, was observed 1 h after the cold shock. At 5 h after the temperature downshift, 199 and 210 genes were up- or down-regulated, respectively. Thus, the relatively small gene set affected initially indicated a targeted acute response to cold shock, whereas extensive metabolic remodeling appeared to take place after prolonged exposure to cold. Genes related to fatty acid biosynthesis, oxidative stress response, and iron uptake and storage were induced, in addition to mechanisms previously characterized as cold-tolerance related in bacteria. Furthermore, several uncharacterized DNA-binding transcriptional regulator-encoding genes were induced, suggesting involvement of novel regulatory mechanisms in the cold shock response of *C. botulinum*. The role of such regulators, CBO0477 and CBO0558A, in cold tolerance of *C. botulinum* ATCC 3502 was demonstrated by deteriorated growth of related mutants at 17°C.

## Introduction

Understanding the mechanisms by which foodborne pathogenic microorganisms cope with stress conditions they encounter in foods is of key importance in designing modern food safety measures. The ability of the anaerobic Gram-positive spore-forming *Clostridium botulinum* to survive, grow and subsequently produce botulinum neurotoxin in foods [1] raises substantial concern over food safety [2], [3].

The approaches to control growth of *C. botulinum* in minimally processed foods differ considerably from those of canned foods, where autoclaving to destroy 12 log-units of spores is the standard control method. In minimal food processing, combinations of environmental hurdles including low water activity, low or high pH, and, most importantly, low storage temperature are used. Exposure of bacteria to sub-lethal stress can result in increased robustness and (cross-)protection towards harsher treatments, thus creating challenges in classical hurdle design in food processing [4]. Hence, identification of mechanisms behind response and adaptation to the environmental hurdles *C. botulinum* may encounter in food processing might provide biomarkers for detection of potentially stress-adapted cells, thus allowing more precise and efficient control.

Exposure to low temperature presents several challenges to the bacterial cell. Upon cold shock, the translational machinery is hampered due to formation of stable secondary mRNA structures and decreased ribosome activity. Moreover, the folding and activity of proteins is slowed down, and the solidification of the cytoplasmic membrane lipids hinders biomolecule transport and other membrane-associated processes [5]. To counter these constraints, the cell elicits a set of targeted defensive responses, namely, the cold-shock response. The genome-wide response to temperature downshift has been extensively characterized in the Gram-positive model organism *Bacillus subtilis* [6], [7]. However, little is known regarding machineries related to sensing and adapting of *C. botulinum* to low temperature.

Of the three cold-shock domain family proteins (Csps) present in the *C. botulinum* type A strain ATCC 3502, CspB has been shown to be the major cold-related Csp [8]. Recently, we have identified two two-component systems (TCS), CBO0366/CBO0365 [9] and CBO2306/CBO2307 [10], that had induced expression upon temperature downshift and an important role in cold adaptation in *C. botulinum* ATCC 3502. Furthermore, a novel role in cold and hyperosmotic stress tolerance of *C. botulinum* was demonstrated for the previously strictly sporulation-associated alternative sigma factor SigK [11].

To gain a more comprehensive picture of the cold-shock response of *C. botulinum* ATCC 3502, we carried out a transcriptomic analysis after a temperature downshift from the optimal 37°C to 15°C. Genes with >2.0 or <**−**2.0 log_2_-fold difference in their transcript levels in comparison to the levels prior to cold shock were considered to be cold-induced or repressed, respectively. We identified differential transcription of 27 genes 1 h after the cold shock, whereas several hundreds of genes were affected 5 h after the cold shock. The most abundant groups of strongly up-regulated genes were involved with fatty acid biosynthesis, transcriptional regulation, and iron transport and storage functions. Additionally, insertional inactivation of the up-regulated *cbo0477* and *cbo0558A*, putatively encoding DNA-binding regulators, resulted in deteriorated cold tolerance, suggesting roles for these regulators in the cold stress response of *C. botulinum* ATCC 3502.

## Results

### Identification of Cold-regulated Genes in *C. botulinum* ATCC 3502

**Figure 1:**
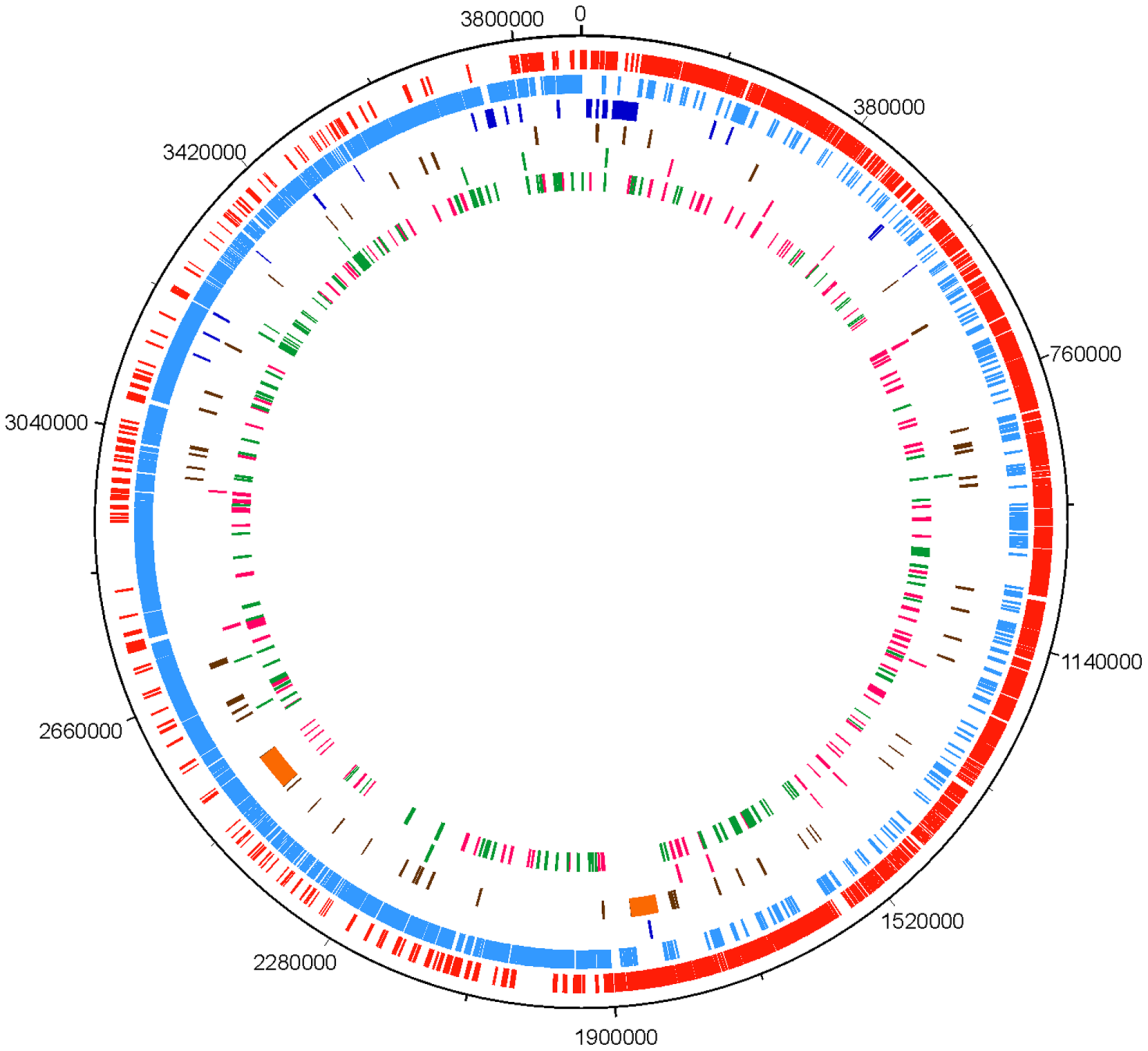
Projection of genes significantly up- or down-regulated upon cold shock in the *C. botulinum* ATCC 3502 genome. From outside to inside: Ring 1, molecular clock of *C. botulinum* ATCC 3502 genome; ring 2, coding DNA sequences of the forward strand (red); ring 3, coding DNA sequences of the reverse strand (cyan); ring 4, ribosomal and transfer RNA (blue); ring 5, pseudogenes (brown) and prophages (orange); ring 6, genes up-regulated (pink) or down-regulated (green) 1 h after the cold shock; ring 7, genes up-regulated (pink) or down-regulated (green) 5 h after the cold shock.

To identify the coding sequences (CDSs) that were significantly induced or repressed upon temperature downshift in *C. botulinum*, we carried out a transcriptomic analysis of the ATCC 3502 strain. The abundance of transcripts in cultures grown at 37°C were compared to those exposed to cold stress at 15°C for 1 h or 5 h using DNA microarrays based on the ATCC 3502 genome [12]. Genes up- or down-regulated by a log_2_-ratio of >2.0 or −<2.0 (false discovery rate [FDR] <0.05), respectively, were considered to be cold-regulated. In total, 199 CDSs were up-regulated 5 h after the cold shock; of these, 16 CDSs showed induction also 1 h after temperature downshift. The analysis revealed 210 CDSs to be down-regulated 5 h after exposure to low temperature; of these, 10 CDSs showed decreased transcript levels 1 h after the cold shock. Additionally, one CDS (*cbo2459*) was down-regulated 1 h but not 5 h after the cold shock. The cold-regulated CDSs were equally distributed among the genome (Fig. 1). The cold-induced and repressed gene set of *C. botulinum* ATCC 3502 is presented in Table S1 Table S1.

### Validation of the DNA Microarray Results with RT-qPCR

**Figure 2:**
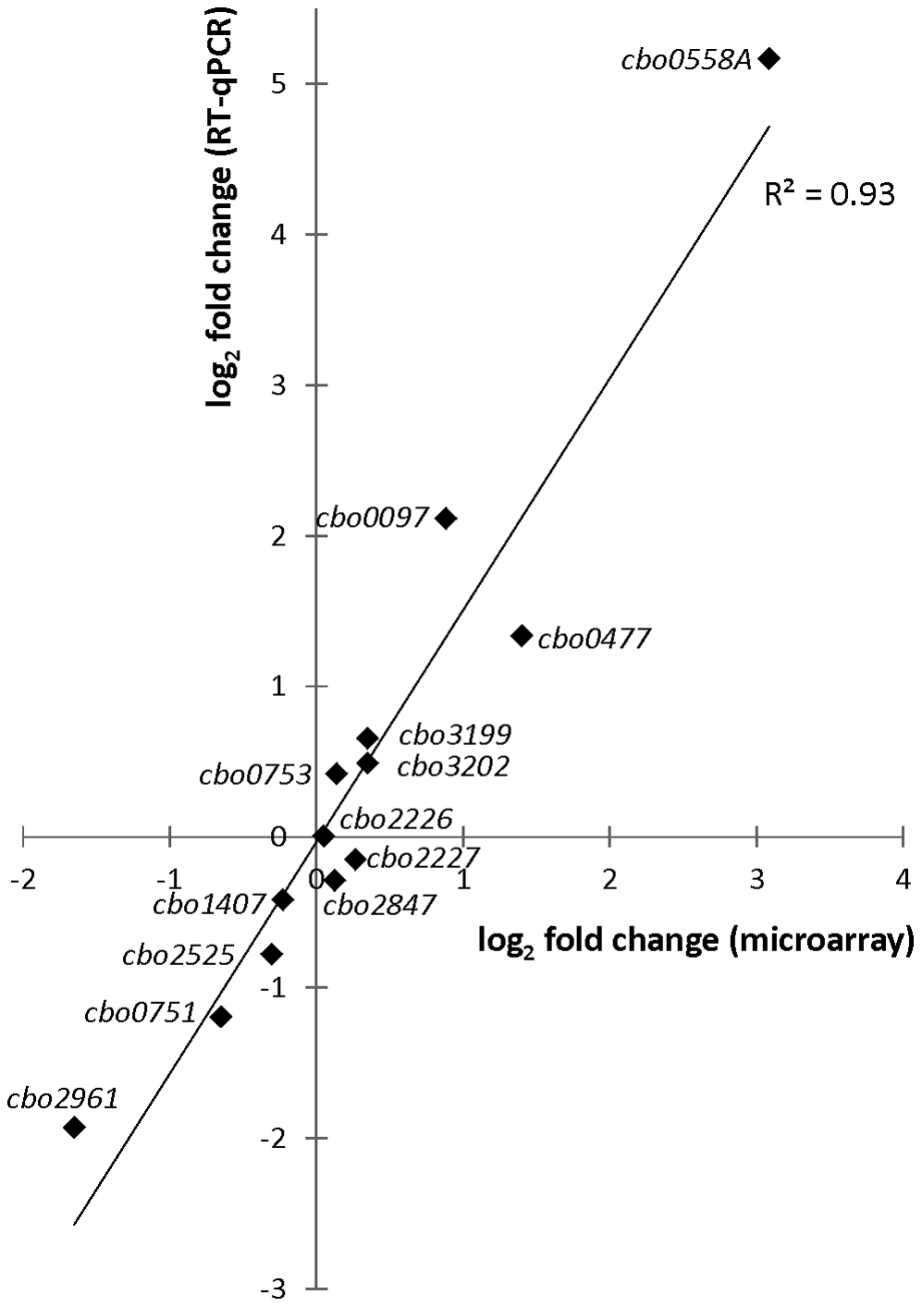
Confirmation of DNA microarray results with quantitative real-time reverse-transcription PCR (RT-qPCR). Log_2_ fold changes of transcript levels measured with DNA microarrays (x-axis) and RT-qPCR (y-axis) in *C. botulinum* ATCC 3502 cultures 1 h after temperature downshift from 37°C to 15°C. 16S *rrn* transcript levels were used as a normalization reference in the RT-qPCR. Linear regression analysis showed an R^2^ correlation value of 0.93 between the microarray and RT-qPCR transcription fold change results.

Quantitative real-time reverse-transcription PCR (RT-qPCR) analysis was carried out to validate the 1-h post-shock expression fold change data obtained from the microarray experiments. Fold changes of transcript levels for genes *cbo0097*, *cbo0477*, *cbo0558A*, *cbo0751*, *cbo0753*, *cbo1407*, *cbo2226*, *cbo2227*, *cbo2525*, *cbo2847*, *cbo2961*, *cbo3199* and *cbo3202* one hour after the cold shock, normalized to 16S *rrn* transcript levels and calibrated to pre-cold-shock transcript levels, were calculated using the Cq values obtained from qPCR runs. The Cq values of the 1-h post-shock transcript levels were obtained in a previous study [13] for genes *cbo0751, cbo0753, cbo1407, cbo2226, cbo2227, cbo2525, cbo2847, cbo3199*, and *cbo3202*; all other data were produced in the current study. In a linear regression analysis between the microarray and RT-qPCR log_2_ fold changes (Fig. 2), a R^2^ correlation value of 0.93 was observed.

### Hierarchical Clustering of Differentially Transcribed Genes and Determination of the Number of Differentially Transcribed Genes in each Functional Category

**Figure 3:**
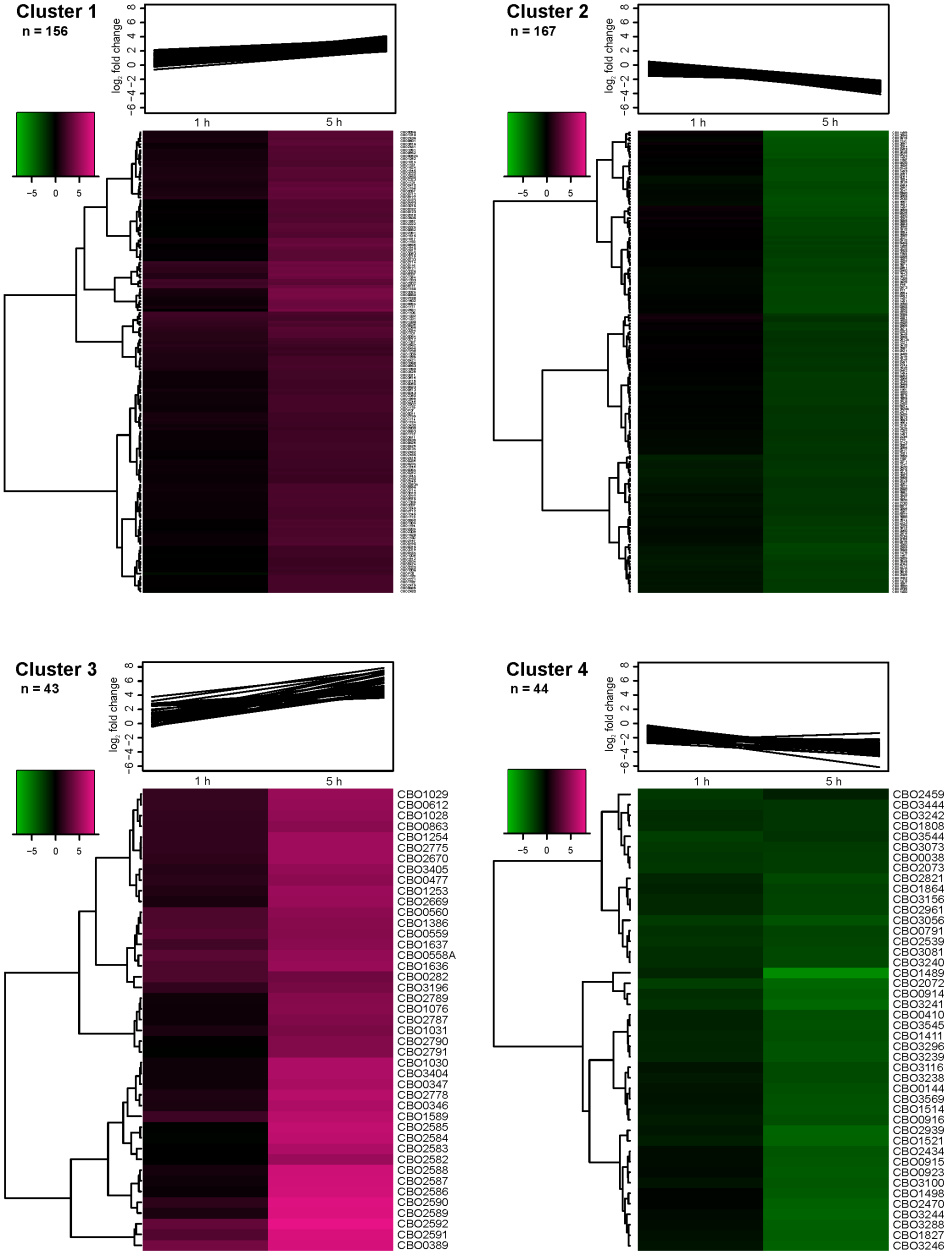
K-means clusters of up- or down-regulated genes 1 h and 5 h after temperature downshift in *C. botulinum* ATCC 3502. K-means clustering of significantly up- or down-regulated genes 1 h and 5 h after temperature downshift from 37°C to 15°C in *C. botulinum* ATCC 3502 into four clusters. The sub-clusters were further subjected to hierarchical clustering.

To identify groups of similar, biologically relevant transcriptional patterns among the cold shock-affected genes, clustering of the data into main- and sub-clusters was performed. Division into main groups was accomplished with k-means clustering method, which is commonly used in microarray data analysis. K-means clustering partitions the data into a user-defined number of clusters based on the expression values included in the analysis. From among the different number of tested k-means clusters, the amount of clusters was chosen to be four as this exhibited biologically relevant partitioning of the genes primarily based on the level of induction or repression at 5 h after the cold shock. Clusters 1 and 2 contained 156 and 167 up- and down-regulated genes, respectively, whereas the number of up- and down-regulated genes in clusters 3 and 4 were 43 and 44, respectively (Fig. 3). The genes within clusters 3 and 4 exhibited markedly stronger up- and down-regulation within the 5-hour experimental time window, respectively, than the genes partitioned into clusters 1 and 2. As the number of differentially transcribed genes at 5 h was substantially greater than at 1 h, the cluster allocation was mostly affected by the 5-h transcript levels and thus failed to notably cluster together genes only affected at 1 h.

Each of the four k-means clusters was separately subjected to hierarchical clustering in order to identify sub-groups of genes with a similar transcriptional pattern within the clusters. The aim of this analysis was to identify putative functional associations within small groups of similarly transcribed genes. Hierarchical clustering grouped together some functional and/or transcriptional units, such as genes involved in anaerobic respiration or regulation of iron metabolism in cluster 1, or genes putatively involved in fatty acid biosynthesis in cluster 3. However, no further association of sub-clusters to functional categories or metabolic pathways could be identified. The k-means main cluster and the hierarchical sub-cluster of each differentially transcribed gene are shown in Table S1 Table S1, along with the functional main- and sub-classes for all the genes represented in the microarray.

**Figure 4:**
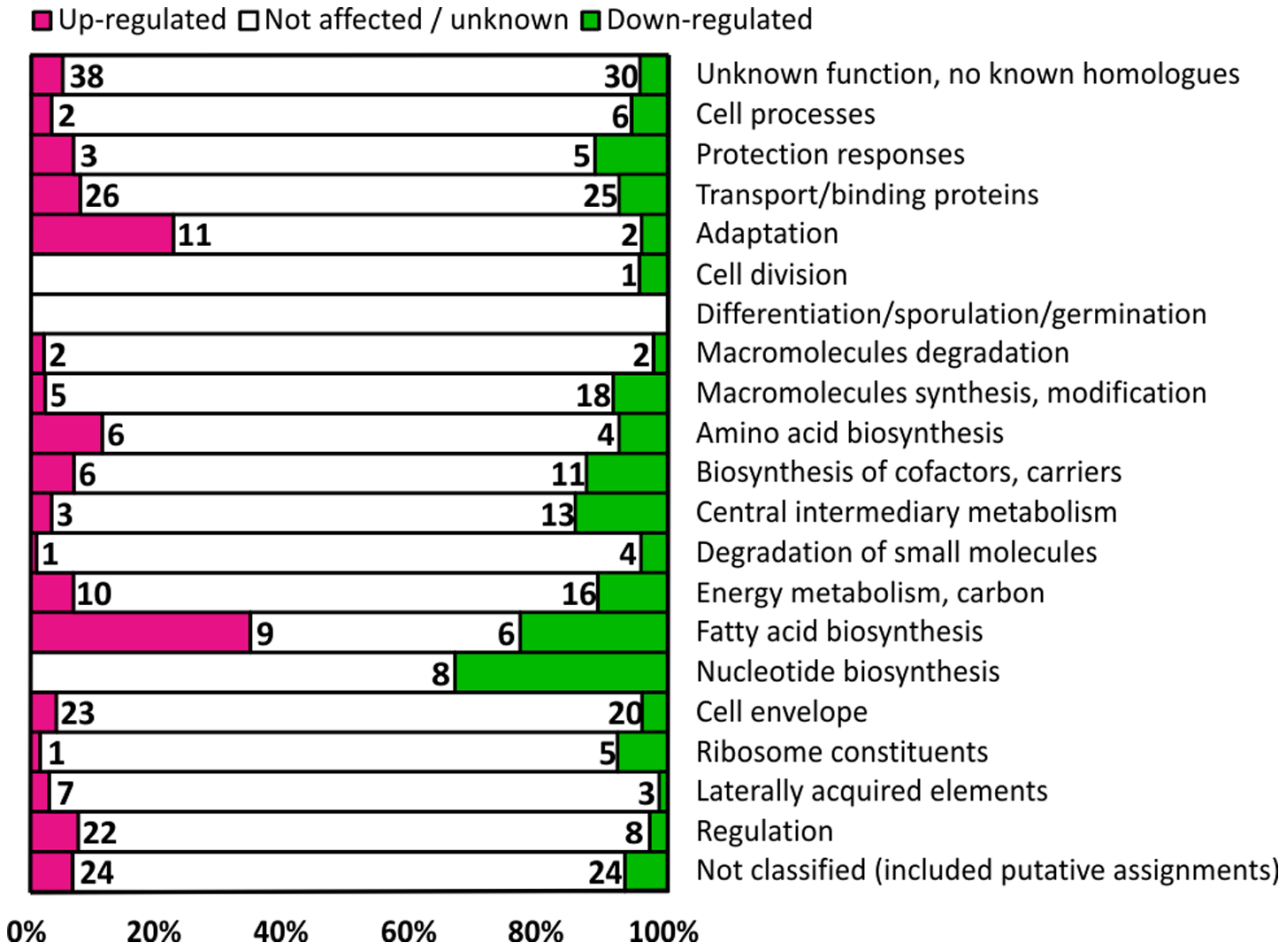
Distribution of significantly up- or down-regulated genes in functional categories. The number of significantly up- or down-regulated and unaffected genes 5 h after temperature downshift from 37°C to 15°C in *C. botulinum* ATCC 3502 divided into functional categories. The bar lengths portray the percentage of affected and unaffected genes of all genes assigned to each functional category. The functional categories were as described in the original annotation of the ATCC 3502 genome [12]. Genes with no expression data available due to poor array hybridization are assigned to the “Not affected/unknown” category.

To gain an insight into genes with similar transcription patterns upon cold shock, the number of up- and down-regulated genes in each functional category was determined. The functional category for each gene was derived from the assignment in the original annotation of the ATCC 3502 genome [12]. This revealed a marked number of down-regulated genes in central metabolism-related categories, and up-regulated genes in adaptation- and regulation-related categories (Fig. 4). The significant components of these functional categories were further combined into biologically relevant metabolic groups when considered necessary.

### The Cold-affected Gene Set 1 h after Temperature Downshift in *C. botulinum* ATCC 3502

A set of 27 genes was up- or down-regulated 1 h after temperature downshift (Table S1 Table S1). Among these was *cbo2802* (*deaD*) putatively encoding a DEAD/DEAH box family RNA helicase, with 4.3-fold up-regulation 1 h after the cold shock. Additionally, *cbo0282* (*cspA*) encoding the cold-shock protein CspA was 6.6-fold up-regulated. The genes *cbo1636* and *cbo1637* predicted to encode the ATP-binding protein and permease A components of the glycine betaine/L-proline ABC transporter OpuC, were induced 4.6- and 6.5-fold, respectively, 1 h after the cold shock.

In addition to the aforementioned annotated genes, several genes without predicted functions were induced. Among these, *cbo2592* and *cbo2591*, the first genes of the putative operon *cbo2592*-*cbo2581*, were 9.2-and 7.0-fold induced 1 h after cold shock, respectively. Additionally, 6.5- to 8.6-fold induction of a putative operon *cbo0558A*-*cbo0560* encoding a DNA-binding transcriptional regulator and components of a putative ABC transporter was observed, as well as 16-fold induction of *cbo0389* encoding a putative amino acid permease.

### The Effects of Prolonged Cold Exposure on the Transcriptome of *C. botulinum* ATCC 3502

The transcription of a large number of genes was markedly affected 5 h after the temperature downshift. The results for these genes are presented in Table S1 Table S1, and elaborated in detail below in the context of their predicted functional categories.

#### Fatty acid metabolism

Of the 26 genes putatively related to fatty acid metabolism, 9 were significantly up-regulated and 6 down-regulated (Fig. 4). The strongest induction of all ORFs represented in the microarray was observed for genes within the putative operon *cbo2592*-*cbo2581*. The first gene therein, *cbo2592*, was 270-fold induced. Functional predictions for several of the operon genes suggest the operon to be involved in fatty acid biosynthesis; however, the rest of the genes within the cluster had poor resemblance to characterized fatty acid metabolism-related mechanisms. Therefore, the definite function of this cluster remains to be confirmed. All six down-regulated genes in this functional category represented the gene cluster *cbo3605*-*cbo3594*. Most genes within this cluster encode proteins that resemble components of well-characterized bacterial fatty acid synthesis pathways. Thus, the down-regulated *cbo3605*-*cbo3594* cluster putatively encodes the primary fatty acid biosynthesis machinery of *C. botulinum* ATCC 3502.

#### Oxidative stress and metal ion homeostasis

Induction of several genes related to countering oxidative stress and maintaining redox balance, including thiol peroxidase (*cbo0501* [*tpx*]), rubredoxin (*cbo1252* [*grxC*]), thioredoxin reductase (*cbo1254* [*trxB1*]), a putative oxidative stress-related operon (*cbo1330*, *cbo1331*, *cbo1332*, *cbo1333*), flavodoxin (*cbo2778*) and oxidoreductase (*cbo3429*), was observed. Furthermore, in the strongly-induced cluster 3 (Fig. 3), genes putatively encoding a ferrous iron transport mechanism (FeoAB) (*cbo1028-cbo1031*, *cbo1076*) were identified. Additionally, induction of another ferrous iron transport gene (*cbo1077* [*feoA*]) was observed in cluster 1 (Fig. 3), along with genes encoding ferric uptake regulator (FUR) family proteins (*cbo2560* and *cbo3220*), a ferritin (*cbo1784* [*ftnA*]), an operon possibly related to metal ion homeostasis (*cbo2224-cbo2222*, *cbo2221*), and two ArsR-type regulators (*cbo0182* and *cbo0477*). Finally, *cbo1795* encoding the DNA-damage related LexA repressor was up-regulated.

#### Genes related to processes of vegetative growth

Cluster 4 (Fig. 3) included strongly-repressed genes mostly involved in vegetative growth. Genes putatively involved in nucleotide synthesis and metabolism (*cbo1808* [*cmk*], *cbo1864* [*deoD*], *cbo2072* [*codA*], *cbo2073* [*codB*], *cbo2821* [*pyrR*], *cbo3081* [*hpt*], *cbo3241-cbo3238* [*pyrBICF*], *cbo3296* [*guaB*], *cbo3544* [*prsA*]), energy metabolism (*cbo1489*, *cbo1498*, *cbo3242*, *cbo3244*, *cbo3288* [*acdA*]), transcription or translation (*cbo2434* [*tsf*], *cbo2939* [*dnaG*]), cobalamin biosynthesis (*cbo0410*, *cbo0914-cbo0916* [*cobQ-cbiB-cobD*]), or cell wall synthesis (*cbo0791* [*dapB*], *cbo1827*, *cbo3100*) were included. In addition, genes putatively co-transcribed with the strongly-affected genes in cluster 4, were identified in the moderately-repressed cluster 2. These included *cbo1499-cbo1500* (*metN2-metI2*), *cbo2938* (*sigA*), *cbo3237-cbo3235* (*pyrKDE*), *cbo3243*, *cbo3287-cbo3286* (*etfB2A2*), *cbo3295* (*guaA*), as well as the cobalt transporters (*cbo0917-cbo0919* [*cbiMNQ*], *cbo3450-cbo3449*) and all the other genes putatively related to cobalamin metabolism (*cbo1492*-*cbo1494* [*cobW*-*cbo1493*-*mtbC*] and *cbo1495* [*mtbB*]).

#### Amino acid metabolism

Ten genes encoding amino acid biosynthesis proteins were differentially transcribed 5 h after the cold shock (Fig. 4). However, based on pathway predictions in the KEGG database (http://www.genome.jp/kegg/), a significant number of differentially transcribed genes from other functional classes (e.g. Transport/binding proteins or Degradation of small molecules) were also associated with the amino acid biosynthetic pathways identified. The results for these genes are therefore also presented here.

In cluster 3 (Fig. 3), *cbo2670-cbo2669* (*argGH*) encoding an argininosuccinate synthase and an argininosuccinate lyase, respectively, which produce arginine (Arg) from L-citrulline, were identified, with respective up-regulation of >46- and 39-fold. However, the genes encoding proteins involved in Arg catabolism, i.e. arginine deiminases (*cbo0065* [*arcA1*] and *cbo1587* [*arcA2*]), carbamate kinase and ornithine carbamoyltransferase (*cbo2594-cbo2593* [*arcCB*]), arginine/ornithine antiporter (*cbo1588* [*arcD*]), and Orn/Lys/Arg decarboxylase (*cbo3116* [*speA*]) producing agmatine from L-arginine, were significantly down-regulated. Additionally, the whole putative transcriptional unit *cbo1882-1877* including the arginine repressor-encoding *cbo1878* (*argR*) was down-regulated.

The glutamate dehydrogenase (*cbo1811* [*gluD*]) converting L-glutamate into 2-oxoglutarate and glutamine synthetase (*cbo3563* [*glnA*]) synthesizing L-glutamine from L-glutamate were down-regulated, whereas glutaminase (*cbo2808* [*glsA*]) converting L-glutamine to L-glutamate was up-regulated. Additionally, *cbo3337-cbo3336* (*lysCA*) and *cbo1912-cbo1911* (*dapF-cbo1911*) contributing to the production of L-lysine (Lys) from diaminopimelate were up-regulated, whereas *cbo1457-cbo1458* (*murEF*), which use diaminopimelate for peptidoglycan biosynthesis, were down-regulated. Furthermore, differential transcript levels for several putative D-proline reductase-related operons were observed. Of these, *cbo2482-cbo2479* (*prdC2*-*cbo2481*-*prdA2*-*cbo2479*) and *cbo2466-cbo2462* (*prdC1*-*cbo2465*-*prdA1*-*cbo2463*-*prdB2*) both encoding electron transfer subunits of proline reductase, subunits of proline reductase, and hypothetical proteins, *cbo2461* (*prdB2*) encoding a subunit of proline reductase, and *cbo2476-cbo2474* (*prdDEF*) encoding D-proline reductase and proline racemase were up-regulated. In contrast, *cbo2489-cbo2488* (*prdC4*-*prdA4*) and *cbo2484-cbo2483* (*prdC3*-*prdA3*) encoding an electron transfer subunit of proline reductase and a partial proline reductase were both down-regulated.

Another strongly up-regulated gene (180-fold) 5 h after the cold shock was *cbo0389* putatively encoding an amino acid permease. Additionally, the transcription of another putatively amino acid permease-encoding *cbo0343* was 4.5-fold induced.

#### Regulatory functions

In total, 30 genes with predicted regulatory roles were identified as differentially transcribed, 22 of which were up- and 8 down-regulated (Fig. 4). Of these, genes with obvious associations with specific functional categories mentioned above are presented under their respective sections.

Several key regulators of central metabolism were affected by temperature downshift. A 7.8-fold induction of the GTP-sensing transcriptional pleiotropic repressor *codY* (*cbo2436*) was observed. Additionally, significant down-regulation was observed for *relA* (*cbo3059*) encoding a GTP pyrophosphokinase that has a function in triggering de-repression of CodY.

Among the cold-induced regulatory genes with no obvious association with structural or functional loci was *cbo0097* predicted to encode a PadR-type DNA-binding transcriptional regulator. This gene, showing a 2 and 10-fold induction 1 h and 5 h after cold shock, respectively, is located within a cluster of three genes, including *cbo0096* encoding a putative uncharacterized membrane protein and *cbo0098* encoding a putative zinc-dependent hydrolase. Similar induction pattern was observed for *cbo0096* and *cbo0097* but not *cbo0098*, suggesting bicistronic transcription of *cbo0097*-*cbo0096*.

The *cbo0477* predicted to encode an ArsR-type transcriptional regulator appears to be co-transcribed with *cbo0478* encoding a putative heavy metal-translocating ATPase. A 2.6-fold induction 1 h after the cold shock was observed for *cbo0477*, the transcript levels further increasing to 30-fold 5 h after the cold shock.

One of the most strongly induced genes 1 h after the cold shock was *cbo0558A*. Its induction at 1 h was 8.5-fold in the microarray experiment, but was shown to be as high as 36-fold in RT-qPCR analysis (Fig. 2). The transcript levels of *cbo0558A* further increased to 35-fold in the microarray data 5 h after the cold shock, which represented the highest level of induction among the regulator-encoding genes. Similar transcriptional patterns were observed for *cbo0558A*, *cbo0559* and *cbo0560*, suggesting a transcriptional link between these genes.

### Inactivation of the Cold-induced Putative Transcriptional Regulators *cbo0477* and *cbo0558A* Results in Deteriorated Cold Tolerance

**Figure 5:**
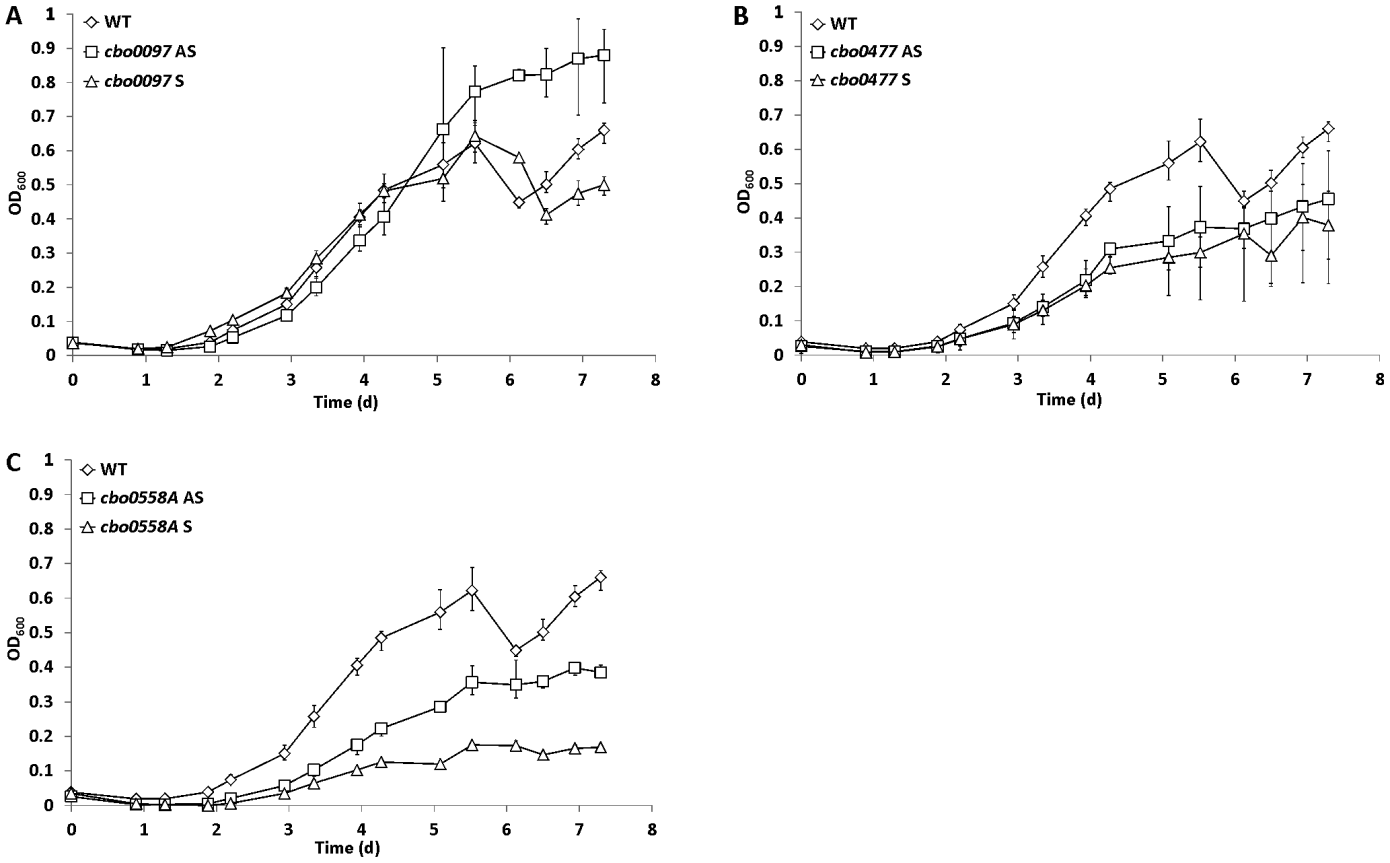
Growth of *C. botulinum* ATCC 3502 wild type, and *cbo0097*, *cbo0477* and *cbo0558A* mutants at 17°C. Growth of *C. botulinum* ATCC 3502 wild type (WT, A-C), *cbo0097* mutant (A), *cbo0477* mutant (B), and *cbo0558A* mutant (C) cultures in TPGY broth at 17°C. Antisense (AS) and sense (S) orientation mutants were constructed for each gene. The error bars represent the minimum and maximum OD_600_ values measured for three independent biological replicates.

To test whether the strongly up-regulated genes encoding DNA-binding regulators have a role in cold tolerance of *C. botulinum* ATCC 3502, we inactivated *cbo0097*, *cbo0477* and *cbo0558A* and compared the growth of such mutants and the wild-type strain at 17°C for 8 days. Inactivation of *cbo0097* did not result in significantly decreased cold sensitivity: The final OD_600_ of the antisense mutant was somewhat higher than that of the sense mutant and the wild-type strain, whereas the sense mutant showed a similar growth pattern to the wild-type strain (Fig. 5A). In contrast, impaired growth at 17°C was observed for all the *cbo0477* (Fig. 5B) and *cbo0558A* (Fig. 5C) mutants. No difference in growth at 37°C was observed between the wild-type strain and any of the mutants (data not shown). Insertional inactivation was performed in two independent sites and orientations to confirm the mutation as the primary cause behind the observed phenotype. The results suggest that the regulatory proteins CBO0477 and CBO0558A have a role in cold tolerance of *C. botulinum* ATCC 3502.

## Discussion

Transcriptomic analysis of the cold-shock response of *C. botulinum* ATCC 3502 revealed induction of metabolic pathways with established cold-related functions, as well as the induction of a large number of uncharacterized genes. Induction of 16 genes was observed already 1 h after the cold shock, indicating a specialized acute response to temperature downshift. All of these 16 genes showed sustained and further increased induction upon extended cold exposure. The data suggest an important role for their encoded mechanisms not only in the rapid cold-shock response, but also in long-term cold adaptation. At the later stage of cold adaptation, marked effects on the transcriptome were observed, suggesting extensive metabolic remodeling upon cold shock.

Analysis of the differentially transcribed genes by k-means clustering suggested biological relevance especially for the strongly-affected clusters 3 and 4. Cluster 3 included up-regulated genes related to previously identified bacterial cold tolerance mechanisms, e.g. compatible solute transport [14], [15], cold shock proteins [5], [8], and a DEAD-box RNA helicase [16], verifying the relevance of the results. Moreover, cluster 4 included down-regulated genes putatively involved in central metabolic and vegetative growth-related processes, such as nucleotide biosynthesis and inter-conversions, consistent with a shutdown of central metabolism associated with cold-shock induced growth arrest and adaptation phase [5].

The DEAD-box proteins have been shown to have a significant role in cold tolerance in a multitude of organisms. They function as RNA helicases and chaperones and thereby counter the effects of increased mRNA stability that hampers translation [16], [17]. Roles in ribosome biogenesis have also been proposed [18]. No reports on the role of DEAD-box helicases in cold tolerance of *C. botulinum* are available. However, a strong induction of DEAD-box helicase genes upon cold stress has been observed at least in *Bacillus cereus* [19], *B. subtilis* [7], *Listeria monocytogenes* [20] and *Yersinia pseudotuberculosis* [21]. These studies used also mutational analysis to support the importance of DEAD-box helicases in cold tolerance of these organisms. The rapid and strong induction of *deaD* upon cold shock suggests a role in cold tolerance in *C. botulinum*; this hypothesis is currently being tested in our laboratory.

*C. botulinum* ATCC 3502 possesses three cold-shock protein (Csp) homologues (*cspABC*) of which *cspB* was suggested to encode the major cold-related Csp [8]. Csps have roles in destabilizing cold-induced mRNA secondary structures thereby facilitating efficient translation [5]. Csps are found in the majority of organisms; however, interestingly, they seem to be absent from (Group II) type E *C. botulinum* genomes [22]. Significant induction of all three Csp-encoding genes already 1 to 30 min after cold shock was observed by Söderholm *et al*. [8]. Similarly, induction of both *cspA* and *cspB* was observed in the current study. Induction of *cspB* was relatively mild (2.2-fold) 1 h after temperature downshift, however, the transcript levels increased to suggest 4.7-fold up-regulation 5 h after the cold shock. No significant change in transcript levels of *cspC* was observed 1 to 5 h after cold shock in the current study, which is in agreement with the findings by Söderholm *et al*. [8].

Among the mechanisms readily induced 1 h after cold shock was the uptake of compatible solutes. The transcript levels of these genes further increased 5 h after the cold shock. In addition, *cbo1131* (*opuC/opuD*) putatively encoding another choline/carnitine/betaine transporter, was significantly up-regulated 5 h after the cold shock. Induction of the high-affinity transport mechanisms for compatible solutes, such as glycine betaine, carnitine, choline and proline, has been linked to osmotic and cold stress in *L. monocytogenes* [14]–[15] and *B. subtilis* [23] and explained by cryo- and osmoprotective functions of these compounds. Cold-induction of related genes in *C. botulinum* ATCC 3502 suggests utilization of compatible solutes as a means to counter cold stress.

A considerable induction of *cbo2592*-*cbo2581*, putatively encoding fatty acid (FA) metabolism-related proteins, was observed upon cold shock. The *cbo2585* encoding a homologue to FabH (3-oxoacyl-[acyl-carrier-protein] synthase 3), responsible for initiation of FA synthesis, is present in this cluster together with two putative acyl-carrier protein genes (*cbo2583* and *cbo2589*). While the genetic arrangement suggests this cluster to be involved in FA metabolism, many of the genes within the cluster have poor homology to previously characterized FA metabolism mechanisms. Moreover, none of the cluster components have been allocated to any specific established pathway in the KEGG database. Thus, the ultimate function of this operon remains to be characterized.

An important negative effect of temperature downshift in the bacterial cell is the solidification of the lipid cell membrane [24]. Bacteria have developed several strategies to restore membrane fluidity and subsequent biological function. These include desaturation of existing membrane FAs by an oxygen-dependent lipid desaturase system [25]–[27], increasing the *de novo* synthesis of unsaturated and branched FAs, and by shortening the acyl chain length of the newly-synthesized FAs [24], [28]. In the Gram-positive *L. monocytogenes* and *B. subtilis*, branched-chain fatty acids (BCFA) are synthesized from branched-chain acyl-coenzyme A (CoA) primers, which are derived from the branched-chain amino acids (BCAA) valine, leucine and isoleucine by branched-chain α-keto acid dehydrogenase (Bkd) enzyme complex [24], [29]–[31]. The FabH of *L. monocytogenes* shows increased affinity to BCAA-CoA primers upon temperature downshift [30], resulting in increased membrane BCFA content and subsequent increase in membrane fluidity.

In addition to *cbo2585*, two other FabH homologues (*cbo0718*, *cbo3604*) were identified in the *C. botulinum* ATCC 3502 genome. They both exhibited down-regulation 5 h after the temperature downshift. The *cbo3604* resides within a cluster of genes with predicted functions in FA synthesis, suggesting *cbo3604* may encode the primary FabH enzyme of *C. botulinum* ATCC 3502. Remarkably, this cluster was significantly down-regulated upon cold shock. This observation, together with the extremely strong cold-induction of *cbo2585* and the adjoining gene cluster, could suggest *cbo2592*-*cbo2581* encodes an alternative cold-triggered FA biosynthesis pathway. Thus, in contrast to the temperature-dependent change in FabH primer preference utilized by *L. monocytogenes* [30], *C. botulinum* could counter cold-induced lipid solidification by switching to a totally different cold-activated FA biosynthesis machinery. Unfortunately, to the authors’ knowledge, the function and substrate-specificity of clostridial FabH enzymes has not been characterized. Furthermore, reports on the utilization of BCAA and the presence of BCFA in *C. botulinum* and in the closely-related *Clostridium sporogenes* are somewhat contradicting [32]–[35]. Thus, further characterization of the FA biosynthesis mechanisms and their role in cold tolerance in *C. botulinum* is warranted.

Several genes with functions predicted in oxidative stress response were significantly cold-induced. Similarly, oxidative stress response was also induced in *B. subtilis* upon cold shock [6] and in *L. monocytogenes* and *Psychrobacter arcticus* during growth at low temperature [36], [37]. Recently, the presence of secondary oxidative stress was demonstrated in *B. subtilis* exposed to a variety of environmental stress conditions [38], [39]. Our observation on the induction of the oxidative stress response upon cold shock strongly supports the hypothesis of secondary oxidative stress as a central player in “general” stress. Therefore, we suggest an important role for induction of the oxidative stress response in countering cold stress in *C. botulinum*.

In addition to mechanisms directly related to oxidative stress, genes related to iron transport and storage were induced upon cold shock, along with two FUR family regulator genes. Induction of genes for ferrous iron transporters and iron-storing ferritins, similar to the gene set induced upon cold stress in our experiment, was observed upon O_2_ stress in *Clostridium acetobutylicum* [40]. Iron possesses a conflictive role upon oxidative stress: ferrous iron feeds the Fenton reaction, resulting in production of toxic hydroxyl radicals [41]. On the other hand, detoxifying and repair enzymes commonly use iron as a cofactor, thus presenting an increased need for iron upon (oxidative) stress [41]. Hillmann *et al*. [40] suggest an induction of iron uptake mechanisms at O_2_ stress in *C. acetobutylicum* to be a response to increased iron demand arising from activation of detoxifying and repair enzymes. A similar goal can thus be hypothesized for the cold-induction of iron uptake in *C. botulinum*. However, the exact role and function of induced iron uptake and storage mechanisms and the FUR family regulators in cold tolerance of *C. botulinum* remain to be elucidated.

Several genes reported to be repressed by the SOS transcriptional repressor LexA in *B. subtilis* [42] were cold-induced in *C. botulinum*. These included *lexA*, *cbo2405* (*recA*), and *cbo2818* (*dinB*, putatively encoding DNA polymerase IV). Upon DNA damage, RecA binds to the resulting single-stranded DNA molecule and facilitates the inactivation of the transcriptional repressor LexA [43]. As a consequence, the LexA-mediated repression of SOS-regulated loci is liberated and the SOS response is activated [43]. Our data suggest activation of the SOS response upon cold shock, implying that direct or indirect DNA damage might result from cold stress in *C. botulinum*. Direct DNA damage can arise from nucleophilic attacks by hydroxyl radicals. As discussed above, the observed induction of oxidative stress-related genes supports the hypothesis of secondary oxidative stress as a player in cold stress. The cold-induction of the SOS response further supports this hypothesis. Considering food-borne pathogens, the increased cross-protective robustness arising from sub-lethal stress due to control measures applied in minimal food processing is of concern. This phenomenon was recently shown in *Bacillus weihenstephanensis*, where cultures grown at 7°C showed increased tolerance towards severe oxidative stress, compared to cells cultured at 30°C [44]. Thus, future research on the role of oxidative stress as a player in the general stress response and in cold-shock response of *C. botulinum* is of special interest.

Upon cold shock, growth is halted until bacteria have adjusted to the changed temperature, with several central metabolic processes being shut down for the adaptation period [5]. Down-regulation of the primary exponential-phase sigma factor SigA (*cbo2938*) is consistent with growth arrest-related shutdown of central metabolism. Almost an equal number of down- and up-regulated genes in the transport/binding proteins, fatty acid biosynthesis and cell envelope classes (Fig. 4) can be explained by changes in cell processes due to the slower growth rate. Among the down-regulated transport/binding protein-encoding genes, ones involved in carbohydrate, cobalt or uracil uptake were identified, i.e. those needed for active growth. However, genes putatively encoding certain amino acid transporting mechanisms were up-regulated. The possible roles of these up-regulated mechanisms in bacterial cold tolerance are discussed below.

Down-regulation of most cobalamin (coenzyme B_12_) synthesis genes and the closely-related cobalt uptake mechanisms was observed. The main function of cobalamin in anaerobic bacteria is thought to be anaerobic fermentation of small molecules (ethanolamine, propanediol or glycerol) and simultaneous generation of an electron sink subsequently balancing redox reactions [45]. In *C. acetobutylicum*, the cobalt- and cobalamin-related genes were observed to be down-regulated upon entry into stationary phase [46]. The down-regulation of cobalamin biosynthesis genes in our experiment can be explained by cessation of growth, or by changes in redox balance-related reactions due to temperature downshift as discussed above.

Extensive remodeling of amino acid uptake and utilization patterns was observed upon temperature downshift. As the catabolism and biosynthesis of Arg are activated and repressed, respectively, by the down-regulated ArgR, the remodeling of the Arg metabolism upon temperature downshift by *C. botulinum* seems to aim in Arg conservation rather than degradation. Arg is an essential amino acid for Group I *C. botulinum* [47], and a high Arg level in the culture medium has been proposed to suppress neurotoxin production and protease activity [48]. However, the apparent shift towards Arg accumulation did not result in down-regulation of the neurotoxin cluster genes: slight up-regulation of *cbo0803-cbo0801* encoding the hemagglutinins was observed, and no significant changes were observed for *cbo0804* (*botR*), *cbo0805* (*ntnh*), or *cbo0806* (*botA*).

As observed for arginine, the transcriptional changes of genes involved in glutamic acid (Glu) metabolism appeared to aim at increasing the amount of Glu within the cell. Furthermore, similar behavior was observed for lysine-associated genes, suggesting Lys accumulation upon cold stress. Finally, the pathway for Stickland-type reduction [49] of D-proline was up-regulated.

In contrast to our findings, studies on *B. subtilis* [6] and *L. monocytogenes* [50] revealed increased expression of BCAA biosynthesis genes at low temperature, without any further cold-triggered effects on amino acid metabolism. In *E. coli*, decreased expression of Arg biosynthesis and degradation genes, as well as of Glu synthase genes was observed at low temperature [5]. Our data suggest that unlike these organisms, *C. botulinum* strives to incorporate Arg, Glu and Lys within the cell after temperature downshift. Bacteria use Arg, Glu and/or Lys decarboxylation to cope with acid stress [51]. Thus, a possible goal for Arg, Glu, and Lys conservation at low temperature could be their use as compatible solutes in *C. botulinum* ATCC 3502. The induction of the D-proline reduction pathway could arise from the probable use of this reaction as an electron sink, thus playing a role in controlling the redox balance of the cell [52]. Induction of the D-proline reductase is thus in agreement with induction of the other redox balance-related genes discussed above.

Strongly-induced transcription of two putative amino acid permeases (*cbo0343* and *cbo0389*) was observed. Amino acid permeases transport (specific) amino acids into the cell. However, neither of the two proteins had significant homology to any characterized proteins of related organisms, thus, the specificity of these transporters remains unknown. However, the observed remodeling of the amino acid metabolism to conserve Arg, Glu, Lys, and Pro suggests that the transporters encoded by *cbo0343* and *cbo0389* could transport these amino acids.

Low temperature induced the transcription of *codY* in *C. botulinum* ATCC 3502. CodY, the global stationary phase regulator, is a key player in the central metabolic processes in several low-GC Gram-positive bacteria [53]. CodY regulates the transcription of over a hundred genes, including itself [54]–[57], generally repressing them during rapid growth and de-repressing them under nutritionally poor conditions (stringent response). Depending on the organism, the CodY repressor activity is enhanced by GTP and/or BCAAs. In stringent conditions, the higher amount of uncharged tRNAs activates RelA-mediated conversion of GTP to guanosine 5′-triphosphate 3′-diphosphate or guanosine 5′-diphosphate 3′-diphosphate (pppGpp and ppGpp, hereafter abbreviated as [p]ppGpp) subsequently lowering the GTP level within the cell and triggering de-repression of CodY-regulated genes [53], [58], [59].

At low temperature, the translational capacity of the cell is hampered due to decreased tRNA synthesis, thus lowering the ratio of uncharged to charged tRNAs, a situation analogous to nutrient-rich conditions. Indeed, several aminoacyl-tRNA genes (*cbo2976* [*selA*], *cbo3503* [*proS2*], *cbo3054* [*aspS*], *cbo3055* [*hisS*], *cbo3265* [*gatB*]) were found to be down-regulated. Furthermore, a decrease in (p)ppGpp levels, with additional effects on the expression of cold shock protein-encoding genes at low temperature, has been reported [60], [61]. Thus, low temperature can partially resemble nutrient-rich conditions in the form of decreased uncharged-to-charged tRNA ratio. This subsequently could decrease RelA activity and result in lower (p)ppGpp levels. However, at low temperature, due to the slower growth rate and down-regulation of purine metabolism genes, the GTP level is possibly also low, resembling stringent conditions. Indeed, *L*. *monocytogenes* was suggested to suffer from amino acid starvation (i.e. stringent conditions) upon cold stress [36]. Thus, a temperature downshift can be hypothesized to possess elements from both nutrient-rich and stringent conditions, resulting in partial de-repression of the CodY regulon.

CodY-mediated control of virulence has been demonstrated in different organisms [56], [62]–[64]. However, as discussed above, no evident changes of transcription were observed for genes involved in virulence upon cold shock in *C. botulinum* ATCC 3502. In *L. monocytogenes*, a similar induction of *codY* at low temperature has been observed without de-repression of certain CodY-regulated genes [50]. These data further support the partial activation of the CodY regulon upon cold shock, suggesting significant plasticity for CodY-mediated regulation in *C. botulinum* under different environmental conditions.

We have previously identified cold-induction of genes encoding the two-component systems (TCSs) CBO0366/CBO0365 and CBO2306/CB02307, and the alternative sigma factor SigK (*cbo2541*) [9]–[11]. Further characterization showed these mechanisms to have a role in cold tolerance of *C. botulinum* [9]–[11]. Significant induction of the *cbo0364-cbo0366* operon was also observed in the present DNA microarray experiment, albeit the 5-h log_2_ fold changes fell slightly outside of the defined cut-off value of 2. However, of all putative TCSs of *C. botulinum* ATCC 3502, this TCS showed the strongest induction upon temperature downshift. A modest induction was also observed for *cbo2306*, while no significant induction was observed for *cbo2307* or *cbo2541*. In our previous RT-qPCR studies, however, the transcription of *cbo2307* or *cbo2541* reached a 3.0 to 4.4-fold induction 5 h after cold shock [10], [11]. The RT-qPCR verification of the DNA microarray data suggests that differences in transcript levels are more modestly interpreted by the microarray experiment than by RT-qPCR. The apparent discrepancy can be attributed to the distinct normalization procedures. However, a good correlation between the microarray and RT-qPCR data was observed and suggests that the main findings discussed are reliable.

The induction of several previously uncharacterized DNA-binding regulatory protein-encoding genes upon cold shock suggested the presence of as yet unidentified cold tolerance mechanisms for *C. botulinum*. Therefore, we further analyzed three rapidly and/or strongly-induced genes representing structurally distinct regulator sub-families. Insertional inactivation of *cbo0477* resulted in increased sensitivity to low temperature, as demonstrated by growth curve analysis (Fig. 5B). The genomic context of *cbo0477* suggests this gene to have a role in metal ion homeostasis, thus having possible implications on cold tolerance as discussed above in context of iron uptake and storage. Moreover, inactivation of the highly-induced *cbo0558A* expectedly resulted in deteriorated growth at 17°C (Fig. 5C), while growth in optimal conditions was not affected. The strong and sustained induction of *cbo0558A* combined with the cold-sensitive phenotypes of mutants of this gene, suggests an important function for the CBO0558A regulator in cold tolerance of *C. botulinum*, and warrants further investigation. A homologue for *cbo558A* is found across Group I *C. botulinum* genomes within an operon of three genes (homologous to *cbo0558A*, *cbo0559* and *cbo0560*), the other two of them putatively encoding components of an uncharacterized ABC transporter system. The induction pattern of *cbo0558A* together with its apparent conservation presents it as an interesting candidate for a biomarker for cold-induced robustness in *C. botulinum*.

While notable cold-induction of *cbo0097* was observed, its inactivation did not result in cold-sensitivity. Therefore, the mechanisms under the regulation of CBO0097 are probably not essential for survival at low temperature, or they may be compensated by other mechanisms.

### Conclusions

Our results suggest a relatively small number of rapidly cold shock-induced genes in *C. botulinum* ATCC 3502, with many previously uncharacterized players. In contrast, the cold-triggered transcriptional changes observed 5 h after the temperature downshift were extensive, indicating a dramatic remodeling of metabolism upon cold adaptation. While several mechanisms that were cold-induced in *C. botulinum* have been previously identified as cold-related also in other bacteria, identification of novel cold-responsive regulators warrants further investigation. Importantly, evidence suggesting a role for secondary oxidative stress and associated protective responses in cold tolerance was obtained, supporting the hypothesis of secondary oxidative stress as an important part of a myriad of stress conditions. Identification of genes with rapid and sustained induction upon cold stress could aid in the discovery of biomarkers for detection of enhanced tolerance and cross-protection against multiple stressors, and should be of profound interest for food safety research.

## Experimental Procedures

### Cold Shock Culture Conditions and RNA Extraction

*C. botulinum* ATCC 3502 was used as a parent strain in this study (Table 1). Cultures for RNA isolation for DNA microarray and RT-qPCR analysis were prepared in our previous study [13]. Briefly, three independent wild-type *C. botulinum* ATCC 3502 cultures (biological replicates) were anaerobically grown in optimal conditions in tryptone-peptone-glucose-yeast extract (TPGY) broth until their optical density at 600 nm (OD_600_) reached approximately 1. The cultures were subjected to temperature downshift to 15°C and incubated anaerobically at 15°C for 5 h. Samples from each culture were anaerobically collected for total RNA isolation immediately before the temperature downshift, and 1 h and 5 h after anaerobic incubation at 15°C. The samples were collected into sterile plastic tubes containing 20% of the sample volume of ice-cold ethanol-phenol (9∶1) solution (Sigma-Aldrich, St. Louis, MO), mixed thoroughly, and incubated on ice for 30 min. Cells were harvested by centrifugation (4°C, 8000×*g*) for 5 min. The cell pellets were immediately frozen to −70°C until RNA extraction.

**Table 1:** Strains and plasmids used in this study.

Strain or plasmid	Relevant properties	Source
Bacterial strains		
* C. botulinum* ATCC 3502	Wild type parental strain	ATCC^1^
* C. botulinum* ATCC 3502 *cbo0097*::intron 124|125AS	Insertional disruption of *cbo0097* at base 124 in antisense orientation, *erm*	This study
* C. botulinum* ATCC 3502 *cbo0097*::intron 84|85S	Insertional disruption of *cbo0097* at base 84 in sense orientation, *erm*	This study
* C. botulinum* ATCC 3502 *cbo0477*::intron 152|153AS	Insertional disruption of *cbo0477* at base 152 in antisense orientation, *erm*	This study
* C. botulinum* ATCC 3502 *cbo0477*::intron 111|112S	Insertional disruption of *cbo0477* at base 111 in sense orientation, *erm*	This study
* C. botulinum* ATCC 3502 *cbo0558A*::intron 121|122AS	Insertional disruption of *cbo0558A* at base 121 in antisense orientation, *erm*	This study
* C. botulinum* ATCC 3502 *cbo0558A*::intron 114|115S	Insertional disruption of *cbo0558A* at base 114 in sense orientation, *erm*	This study
Plasmids		
pMTL007C-E2	ClosTron plasmid, *catP*, L1.LtrB intron with *ermB* RAM, constitutive intron expression under *fdx* promoter	University of Nottingham [77]
pMTL007C-E2:: *cbo0097*-124|125AS	pMTL007C-E2 with L1.LtrB retargeted to base 124 of *cbo0097* in antisense orientation	This study
pMTL007C-E2:: *cbo0097*-84|85S	pMTL007C-E2 with L1.LtrB retargeted to base 84 of *cbo0097* in antisense orientation	This study
pMTL007C-E2:: *cbo0477*-152|153AS	pMTL007C-E2 with L1.LtrB retargeted to base 152 of *cbo0477* in sense orientation	This study
pMTL007C-E2:: *cbo0477*- 111|112S	pMTL007C-E2 with L1.LtrB retargeted to base 111 of *cbo0477* in antisense orientation	This study
pMTL007C-E2:: *cbo0558A*-121|122AS	pMTL007C-E2 with L1.LtrB retargeted to base 121 of *cbo0558A* in sense orientation	This study
pMTL007C-E2:: *cbo0558A*- 114|115S	pMTL007C-E2 with L1.LtrB retargeted to base 114 of *cbo0558A* in antisense orientation	This study

1 ATCC, American Type Culture Collection.

The cell pellets were thawed on ice for 5 min and used for RNA extraction using the RNeasy Mini Kit or RNeasy Midi Kit (Qiagen GmbH, Hilden, Germany) according to the manufacturer’s instructions. The cells were lysed with a solution containing 25 mg/ml lysozyme (Sigma-Aldrich, St. Louis, MO, USA) and 250 IU/ml mutanolysin (Sigma-Aldrich) in Tris-EDTA buffer (pH 8.0, Fluka BioChemica, Buchs, Switzerland) and agitated at 37°C for 30 min. To ensure efficient removal of all genomic DNA, an additional DNase treatment was carried out using the DNA-free Kit (Life Technologies, Carlsbad, CA) according to manufacturer’s instructions.

The RNA yield and purity (A_260_/A_280_) were checked using the NanoDrop ND-1000 spectrophotometer (Thermo Fisher Scientific Inc., Waltham, MA). The A_260_/A_280_ ratio was >2.0 for all samples. Integrity of RNA was confirmed with miniaturized gel electrophoresis in the Agilent 2100 Bioanalyzer (Agilent Technologies Inc., Santa Clara, CA). The RNA integrity number was >9.2 for all RNA samples.

### cDNA Synthesis

For DNA microarray analysis, a total of 2 µg of each RNA sample was reverse-transcribed into cDNA and simultaneously labeled with fluorescent dyes. In brief, each 30-µl labeling reaction contained 0.2 µg/µl of random hexamers (Life Technologies), 0.01 M DTT (Life Technologies), 1.3 U/µl ribonuclease inhibitor (Life Technologies), 0.5 µM dATP, dTTP and dGTP, 0.2 µM dCTP, 1.7 nmol of Cy-3 or Cy-5 labeled dCTP (GE Healthcare, Pittsburgh, PA), 13 U/µl of SuperScript III reverse transcriptase (Life Technologies), and appropriate buffer (1 × First Strand Buffer, Life Technologies), and was incubated at 46°C for 3 h. RNA hydrolysis and reaction inactivation were performed by addition of 0.5 mM EDTA and 10 µl of 0.1 M NaOH and incubation at 70°C for 15 min. The reactions were subsequently neutralized by addition of 10 µl of 0.1 M HCl. The cDNA was purified with QIAquick PCR Purification Kit (Qiagen), with final elution volume of 40 µl. The cDNA concentration of each sample was measured with NanoDrop.

For RT-qPCR, a total of 500 ng of each RNA sample was used for cDNA synthesis using the DyNAmo cDNA Synthesis Kit (Thermo Fisher Scientific) as instructed by the manufacturer. Each 20-µl reaction, containing 15 ng/µl of random hexamers, 10 IU of M-MuLV RNase H+ reverse transcriptase solution (Thermo Fisher Scientific), and appropriate buffer containing dNTP and MgCl_2_ in a final concentration of 5 mM (1 x, Thermo Fisher Scientific), was incubated at 25°C for 10 min and 37°C for 30 min, then inactivated at 85°C for 5 min and finally chilled to 4°C. Two replicate RT reactions were made for each RNA sample. The cDNAs were stored at −20°C until performing RT-qPCR.

### Transcriptomic Analysis with DNA Microarrays

Array hybridizations for samples collected at mid-logarithmic growth at 37°C and 1 h post-cold were performed in our previous study [13], and samples collected 5 h post-cold shock were similarly hybridized in this study. In brief, *in situ* -synthesized Custom Gene Expression Microarrays (8x15K, Agilent Technologies) were designed to cover 3641 chromosomal (out of the total of 3648) and all 19 plasmid-borne open reading frames (ORF) in the ATCC 3502 genome [12]. Depending on the length of ORF, a total of 3 to 14 60-mer oligonucleotide probes were designed for each ORF.

For array hybridizations, the three biological replicate samples for each time point were labeled with either Cy3 or Cy5 as described above; for each time point, two of the samples were labeled with one dye and one with another. A total of 300 ng of Cy3-labelled cDNA and 300 ng of Cy5-labelled cDNA were mixed into a final volume of 18 µl, and 0.1 mg/ml salmon sperm DNA (Life Technologies) was added into the reaction. The DNA was denatured at 95°C for 2 min and chilled on ice, and finally mixed with blocking agent (Hi-RPM GE Hybridization Kit, Agilent Technologies) and hybridization buffer (Hi-RPM GE Hybridization Kit, Agilent Technologies) as instructed by the manufacturer. A volume of 50 µl of the mix was pipetted onto the DNA microarrays. The arrays were hybridized at 65°C overnight, and washed as instructed (Gene Expression Wash Buffer Kit, Agilent Technologies).

The slides were scanned (Axon GenePix Autoloader 4200 AL, Molecular Devices LLC, Sunnyvale, CA) at 532 and 635 nm using a 5-µm resolution. Image processing was done using the GenePix Pro 6.0 software (Molecular Devices) and data analysis with *R limma* package [65]. Foreground and local background intensities of each spot were characterized by the mean and median pixel values of the spot, respectively. Local background was subtracted from the foreground signal using the “normexp” method with offset value of 50 [66]. The Cy3 and Cy5 channels were converted into a logarithmic (log_2_) scale and normalized using quantile normalization [67]. The raw microarray expression data collected at 37°C and 1 h post-shock have been previously deposited in the Gene Expression Omnibus under accession number GSE26587 [13]. The 5-h raw data and the normalized log_2_-ratios of differential transcription 1 h and 5 h after the cold shock compared with transcription before the shock have been deposited under accession number GSE51465. Statistical analysis was performed to find differentially transcribed genes at 1 h and 5 h after the temperature downshift, in relation to transcript levels before cold shock. The analysis was done separately for each probe to control variation within a coding sequence. Moderated t-test with empirical Bayes variance shrinkage was applied to each probe on the array and the resulting *p*-values were converted into false discovery rate (FDR) values [68]. For each ORF, the probe with median unmodified *p*-value for the difference in transcript levels was chosen to represent the ORF. Of these, ORFs with FDR <0.05 were subsequently considered to have a significant difference in transcript levels. Finally, significantly differentially transcribed ORFs with a log_2_ fold change <−2.0 or >2.0 at either time point were considered to be included in the cold-regulated gene set.

The identified ORFs were further analyzed by clustering methods with the *R stats* and *gplots* packages. The data were divided into 4 to 12 clusters by k-means clustering (Euclidean distance, Hartigan-Wong method [69], [70]). Each k-means sub-cluster was subjected to hierarchical clustering (Euclidean distance, Ward’s method [71]), and the resulting dendrograms were visualized as heatmaps. Additionally, a log_2_ fold change <−1.0 or >1.0 (FDR <0.05) was accepted for the ORFs belonging to the same transcript or functional category with the cold-regulated ORFs when considering induction or repression of metabolic pathways; these genes, however, were not included in the clustering. The number of up- or down-regulated genes in each functional category (Table S1 Table S1) was determined. The category assigned for each gene was derived from the original annotation of the ATCC 3502 genome [12].

### RT-qPCR

To validate the differences of transcript levels observed in the DNA microarray experiments, RT-qPCR was performed for selected genes (*cbo0097*, *cbo0477*, *cbo0558A*, *cbo0751, cbo0753, cbo1323*, *cbo1407, cbo2226, cbo2227, cbo2304*, *cbo2525, cbo2847, cbo2961*, *cbo3197*, and *cbo3202*) on samples used for microarray hybridizations. The Cq values of the 1-h post-cold-shock samples for genes *cbo0751, cbo0753, cbo1407, cbo2226, cbo2227, cbo2525, cbo2847, cbo3199*, and *cbo3202* were obtained in a previous study [13]. The Cq values of the pre-cold shock samples for all the selected genes, and for the 1-h post-cold-shock samples for genes *cbo0097*, *cbo0477*, *cbo0558A, cbo1323*, *cbo2304*, and *cbo2961*, were obtained in the current study. The DyNAmo Flash SYBR Green qPCR Kit (Thermo Fisher Scientific) was used according to the manufacturer’s instructions to set up two replicate qPCR reactions for each cDNA sample. Each reaction consisted of 1 × Master Mix (Thermo Fisher Scientific), 0.5 µM of forward and reverse primer (Table S2), and 4 µl of 1∶20 (*cbo0097*, *cbo0477*, *cbo0558A*, *cbo1323*, *cbo2304*, *cbo2961*, *cbo3197*, *cbo3202*), 1∶10^3^ (*cbo0751, cbo0753, cbo1407, cbo2226, cbo2227, cbo2525, cbo2847*), or 1∶10^5^ (16S *rrn*) diluted cDNA in a total volume of 20 µl. The reactions were performed in the Rotor-Gene RG3000 thermal cycler (Qiagen). The program consisted of an initial heating step of 7 min 95°C to activate the DNA polymerase, followed by 40 cycles of denaturation at 95°C for 10 s and annealing and extension at 60°C for 20 s, except for primers *cbo0751, cbo1407, cbo2226,* and *cbo2847*, where the program consisted of an initial heating step of 7 min 95°C, and 40 cycles of denaturation at 95°C for 10 s, annealing at 55°C for 15 s followed by extension at 72°C for 15 s. The 1∶10^5^ diluted no-RT controls were analyzed using the 16S *rrn* primers and reaction conditions described above, and no-template controls were included in each run.

The relative quantification of target gene transcript levels at 1 h after temperature downshift, normalized to reference gene (16S *rrn*) transcript levels and calibrated to the samples taken before the cold shock, were calculated by using the Pfaffl method [72]. The method takes into account individual primer efficiencies, which were obtained from RT-qPCR standard curves prepared from serial dilutions of pooled cDNA samples. The Cq values measured for 16S *rrn* remained stable throughout the experiment, supporting its use as a reliable normalization reference gene in the conditions studied. 16S *rrn* is the only previously reported reference gene for *C. botulinum* [73], [74]; no other suitable reference genes have been reported.

### Mutant Construction

Insertional knock-out mutants for *cbo0097*, *cbo0477* and *cbo0558A* with intron insertions in two different sites and orientations were constructed using the ClosTron technology [75]–[77]. ClosTron pMTL007C-E2 vectors with *de novo* synthesized intron targeting regions (DNA2.0 Inc., Menlo Park, CA) were used for the mutations. The intron insertion sites and orientations for the mutant strains are presented in Table 1; primer sequences used to confirm the insertion site and orientation are presented in Table S2. Single intron insertion was confirmed by Southern blotting with a probe targeted to the inserted sequence as described [78].

### Growth Experiments

For evaluation of growth at low temperature, three biological replicate cultures of wild-type *C. botulinum* ATCC 3502, and *cbo0097*, *cbo0477* and *cbo0558A* mutants were anaerobically grown in TPGY broth at 37°C for 12 h or at 17°C for 7 days as described [11]. The temperature of 17°C was used to avoid the notable growth variation observed in the wild-type cultures at 15°C, a temperature close to the minimum growth temperature of *C. botulinum* ATCC 3502 [79]. The OD_600_ of the cultures grown at 37°C was measured anaerobically at 1-h intervals, and twice daily for cultures grown at 17°C. Growth curves were constructed by plotting the OD_600_ of each culture against time.

## Supporting Information

Table S1 **Transcript fold changes of** ***C. botulinum*** **ATCC 3502 1 h and 5 h after temperature downshift from 37°C to 15°C.** **Tab 1** **, All normalized transcript fold changes; Tabs 2 to 5, k-means clustering of significantly (FDR<0.05) >2.0 or <**–**2.0 log_2_ fold-affected genes; Tab 6, genes significantly (FDR<0.05) >2.0 or <−2.0 log_2_ fold-affected at 1 h after cold shock; Tab 7, genes significantly (FDR<0.05) >2.0 or <−2.0 log_2_ fold-affected at 5 h after cold shock; Tab 8, functional categories.**
(XLSX)

Table S2 **Oligonucleotide primers used in the study.**
(XLSX)
